# Impact of COVID-19 on Fracture Incidence in Germany: A Comparative Age and Gender Analysis of Pre- and Post-Outbreak Periods

**DOI:** 10.3390/healthcare11152139

**Published:** 2023-07-26

**Authors:** Tizian Heinz, Moritz Wild, Annette Eidmann, Manuel Weißenberger, Dominik Rak, Alexander Johannes Nedopil, Maximilian Rudert, Ioannis Stratos

**Affiliations:** 1 Department of Orthopaedic Surgery, Koenig-Ludwig-Haus, University of Wuerzburg, 97074 Wuerzburg, Germanym-wild.klh@uni-wuerzburg.de (M.W.); a-eidmann.klh@uni-wuerzburg.de (A.E.); d-rak.klh@uni-wuerzburg.de (D.R.);; 2 Department of Orthopedic Surgery, Orthopedic Surgeon Adventist Health Lodi Memorial, Lodi, CA 95240, USA; ajnedopil@ucdavis.edu

**Keywords:** COVID-19, fracture incidence, age and gender analysis, pre- and post-outbreak comparison, fragility fractures, linear regression

## Abstract

In March 2020, Germany imposed a nationwide lockdown to curb the spread of COVID-19, prompting questions about the impact on the incidence of common fractures. This study examined 15 fracture types in pre-outbreak (2010–2019) and post-outbreak (2020–2021) periods, using data categorized by age (18–64 years, >65 years) and sex (male, female). Linear regression assessed annual growth rates, and mean fracture numbers were compared across periods for significant differences. Results indicated a positive correlation between fracture incidence rates and time for various types, such as cervical, thoracic, lumbar, and pelvic spine fractures, rib fractures, femoral neck, pertrochanteric femur, femoral shaft, and ankle fractures. Frequencies of proximal humerus, distal radius, femoral neck, pertrochanteric femur, femoral shaft, and ankle fractures in 2020 and 2021 were within predicted ranges from previous years. However, rib fractures and spinal fractures (cervical, thoracic, lumbar, and pelvic spine) occurred less frequently during this time. Notably, this study found a consistent decline in most fracture types for individuals aged 18–64 after the pandemic’s onset, while the fracture incidence of hip fractures, often referred to as fragility fractures, for those over 65 remained unchanged. Fibula fractures showed the most considerable decrease in both age groups. In conclusion, the COVID-19 pandemic substantially impacted fracture incidence, with lower rates among individuals under 65 and unchanged fragility fractures in the elderly population.

## 1. Introduction

In March 2020, a drastic nationwide lockdown was declared in Germany to slow down the spread of the emerging acute respiratory syndrome coronavirus 2 (COVID-19) [1]. Consequently, unnecessary travel was banned, schools and public places were closed, and in some regions, even strict curfews were enforced [2]. This fundamental disruption of public life was accompanied by an unprecedented change in the habits and daily activities of the population. The 99% decrease (from 14,066,000 airline passengers in January 2020 to 266,000 passengers in April 2020) in air traffic at German-based airports following the lockdown illustrates the comprehensive scope of the nationwide restrictions imposed [3].

Along with the pandemic and subsequent lockdown, new challenges arose for the public health sector in Germany. The increasing number of mental and emotional disorders resulting from the burdensome restrictions became the focus of scientific attention [4]. Meanwhile, the aspect of physical injuries and their changes during the pandemic was often overlooked. Nevertheless, fractures occur frequently, and their treatment causes a major burden on the public health sector in most Western countries [5,6,7].

Nationwide established registries throughout the European nations allow for the identification of commonly encountered fractures. Among them are distal radius fractures, femoral fractures, including the trochanteric region and neck of the femur, ankle fractures, vertebral, pelvic ring, proximal humerus, and rib fractures [4,5,6,8]. Several differences have been reported regarding the distributional patterns with a strong prevalence of vertebral and proximal femur fractures in the elderly population, contrary to ankle fractures predominantly being found in younger patients [5,9,10].

This study investigated the incidence of 15 common fractures in the adult German population. The primary objective was to explore and report the potential influence of the COVID-19 pandemic on fracture incidence in Germany by comparing the pre-pandemic period (from 2010 to 2019) to the post-pandemic period (from 2020 and 2021).

## 2. Materials and Methods

Part of Dataset 23131–0002 from the German Federal Statistical Office was used to identify the most common fracture types among adults residing in Germany. The data set was prepared and truncated according to our specifications by the Federal Statistical Office. The dataset contains information about hospital patients in Germany, categorized by the year of data collection, gender, age groups, and one primary diagnosis related to hospital stays based on the ICD-10. These data were further classified into two age groups, distinguishing between 18–64 years and >65 years, as well as between male and female patients over the period from 2010 to 2021. The period from 2010 to 2019 was considered the pre-outbreak period, while 2020 and 2021 were defined as the post-outbreak period. The pre-outbreak period was chosen to be longer than the post-outbreak period in order to reduce the variation of the fracture incidence over time. The post-outbreak period had to be limited to two years, as no further data were available at the time of this study. Furthermore, a two-year pre-outbreak (2018 and 2019) period was also considered for statistical analysis. From the data set 12111-0004 of the Federal Statistical Office, the population of Germany was determined by year and by gender. These data were used to calculate the incidence of fractures.

The following fifteen common fracture groups were identified using the ICD-10 fracture codes: fractures of the neck of the femur (S72.0); pertrochanteric femur (S72.1), distal radius (S52.5, S52.6, and S52.7); proximal humerus (S42.2); lumbar spine and pelvis (S32.0, S32.1, S32.3, S32.4, S32.7, S32.8, S33.1, S33.2, and S33.3); fibula (S82.11, S82.21, S82.31, S82.4, S82.6, S82.7, S82.81, and S82.82); ribs (S22.3 and S22.4); thoracic spine (S22.0, S22.1, and S23.1); clavicle (S42.0); proximal tibia (S82.1); femoral shaft (S72.3); subtrochanteric femur (S72.2); humeral shaft (S42.3); and cervical spine (S12.0, S12.1, S12.2, S12.7, S12.9, and S13.1).

Between 2010 and 2021, a dataset including fractures from patients over the age of 18 was analyzed. The dataset revealed a total of 6,755,870 fractures, consisting of fractures of the neck of the femur (906,507; ♂/♀: 0.47/1), pertrochanteric femur (824,301; ♂/♀: 0.41/1), distal radius (1,007,290; ♂/♀: 0.27/1), proximal humerus (710,986; ♂/♀: 0.33/1), lumbar spine and pelvis (928,303; ♂/♀: 0.52/1), fibula (600,695; ♂/♀: 0.77/1), ribs (430,405; ♂/♀: 1.32/1), thoracic spine (315,758; ♂/♀: 0.57/1), clavicle (252,036; ♂/♀: 2.53/1), proximal tibia (220,941; ♂/♀: 0.65/1), femoral shaft (150,493; ♂/♀: 0.54/1), subtrochanteric femur (149,549; ♂/♀: 0.44/1), humeral shaft (141,767; ♂/♀: 0.50/1), and cervical spine (116,839; ♂/♀: 1.11/1).

Statistical analysis was performed using linear regression analysis in GraphPad Prism (version 9.5.1), including 99% confidence intervals and a graphical representation. The F-test of overall significance was used to test whether the linear regression model was significantly different from the intercept-only model. To compare the mean fracture rate of different fracture types before and after the onset of the pandemic, either a *t*-test or a Mann–Whitney U test was utilized. The data were examined to evaluate normality and equality of variance. If both equal variance and normality were satisfied, a *t*-test was employed. If the data violated either assumption, a Mann–Whitney U test was used. The mean percentual change in the fracture volume for the pre- and post-outbreak period was calculated for each fracture type, including a comparison of age within the genders and vice versa, as well as an analysis of the numbers for each fracture type from the pre- to post-outbreak period within the gender and age groups. The level of significance was set at *p* ≤ 0.01 for the statistical analysis.

## 3. Results

In 2019, the fifteen most frequent fractures among adults in Germany were lumbar spine and pelvis fractures (83,706 cases), distal radius fractures (82,155 cases), femoral neck fractures (81,594 cases), pertrochanteric femur fractures (73,798 cases), proximal humerus fractures (61,674 cases), rib fractures (38,915 cases), fibula fractures (35,703 cases), thoracic spine fractures (28,497 cases), clavicle fractures (23,622 cases), proximal tibia fractures (19,619 cases), femoral shaft fractures (14,154 cases), subtrochanteric femur fractures (13,831 cases), humeral shaft fractures (12,498 cases), and cervical spine fractures (11,558 cases).

**Figure 1:**
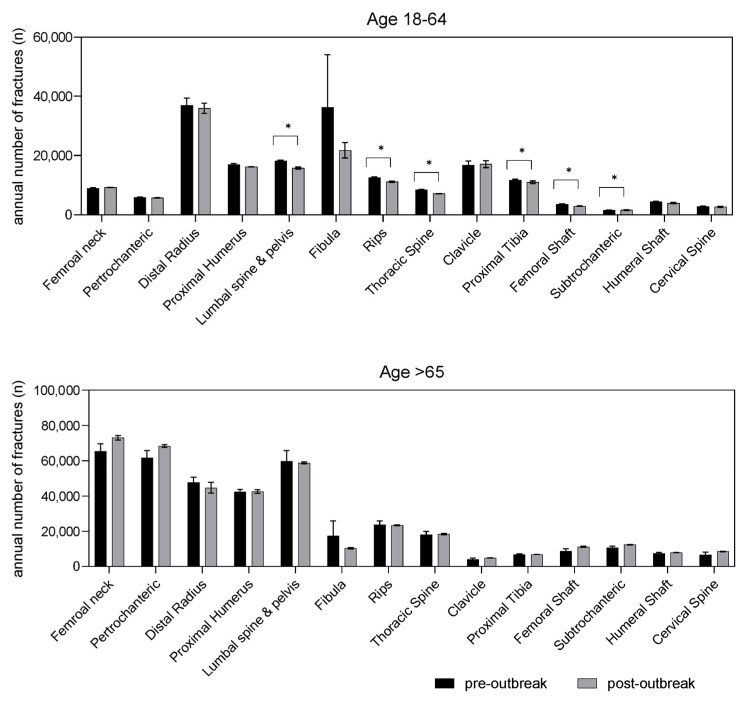
Mean as well as standard deviation per year of fractures for the pre- and post-outbreak period for patients 18–64 years (upper panel) and for patients > 65 years (lower panel). The mean fracture values of the different fracture types before and after the onset of the pandemic outbreak were analyzed via a *t*-test (* *p* ≤ 0.01). Data are given as mean ± standard error of the mean. The relative change in the fractures and the level of significance are displayed in Table 1.

A significant variation in fracture incidence was observed between the pre-outbreak period of 2010–2019 and the pandemic period of 2020–2021 for multiple fracture types (Figure 1). For younger individuals, an overall decline with a significant decrease in rib, thoracic spine, lumbar spine/pelvis, proximal tibia, subtrochanteric, and femoral shaft fractures (Figure 1, Table 1) was observed. In the population group aged > 65 years, the pandemic led to a moderate decrease only in fractures of the fibula, while a tendency toward an increase was observed in fractures of the proximal femur (fractures of the femoral neck, per- and subtrochanteric fractures) and of the femoral shaft (Table 1, Figure 1). When further dividing the population by sex (male and female) and age, significant variance in fracture incidence was still noticeable for the pre- and post-outbreak periods (Table 2). The younger female population experienced significantly fewer fractures post-outbreak than pre-outbreak for the lumbar spine and pelvis, ribs, thoracic spine, and femoral shaft. Younger males had a higher pre-outbreak incidence for distal radius, proximal humerus, lumbar spine and pelvis, fibula, ribs, thoracic spine, clavicle, femoral shaft, and humeral shaft fractures. No fracture type increased after the outbreak for younger males. In contrast, elderly females had more post-outbreak fractures of the femoral neck, as well as subtrochanteric and the femoral shaft fractures, while only fibula fractures were more common pre-outbreak (Table 2). Elderly males also had more post-outbreak femur fractures, including femoral neck, pertrochanteric, subtrochanteric, and femoral shaft fractures.

When narrowing the pre-pandemic period to two years, a significant variance of annual cases was still noticeable, with a significant decline, especially in rib fractures, lumbal spine humeral shaft, and thoracic spine fractures, irrespective of age and gender. There was also a significant decline in proximal tibia fractures for the males aged 18 to 64 years (Table 3). However, changes in fracture incidence between the pre- and post-outbreak periods were smaller when compared with the increased pre-pandemic time period (2010–2019) due to reduced variance of fracture incidence for the increased pre-pandemic period.

**Figure 2:**
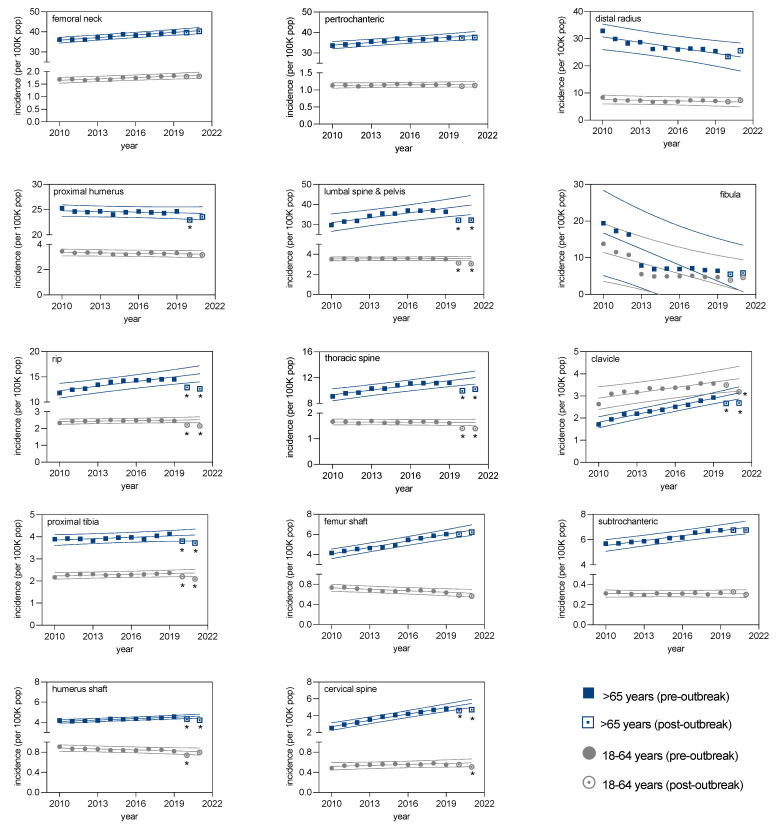
Number of fractures for the years from 2010 to 2021. The years are indicated on the x-axis, and the incidence (number of documented cases per 100,000 citizens) on the y-axis. Calculated are the regression lines (straight lines) as well as the 99% prediction intervals (dotted lines). The prediction intervals are generated by the dataset from 2010 to 2019 and are extrapolated for 2020 and 2021. Blue color is used for patients >65 years of age, and gray color for patients between 18 and 64 years. Full squares and circles are used for the pre-outbreak period; open squares and circles are used for the post-outbreak period. The (*) indicates that the value for the year is outside the 99% prediction interval.

For the pre-pandemic period, linear regression analysis was used to model the incidence rate over time (Figure 2). There was a significant linear relationship for all fracture types in the elderly population, with most fracture types indicating a significant upward trend (*p* = 0.000–0.006), except for distal radius and fibula fractures, which experienced a significant downward trend. Extrapolation of the pre-pandemic 99% prediction intervals to the post-outbreak period showed a significant drop in the observed fracture incidence in the elderly for the (1) proximal humerus, (2) lumbar spine/pelvis, (3) ribs, (4) thoracic spine, (5) clavicle, (6) proximal tibia, (7) humeral shaft, and (8) cervical spine fractures (Figure 2).

## 4. Discussion

As a major finding of this study, the distribution and frequency of commonly encountered fractures have seen significant changes with the onset of the COVID-19 pandemic in Germany. Overall, there was a noticeable decrease in the incidence of the most common fracture types when compared with the average fracture numbers before the pandemic, particularly among younger individuals. This reduction seems consistent, given that the pandemic led to the promotion of new governmental regulations, including home isolation and travel restrictions, which, in turn, triggered significant changes in social behavior, population mobility, and leisure activities. Similarly, various research groups reported an overall decline in trauma-associated surgeries and orthopedic consultations during the COVID-19 period [1,2,3,4,6]. While the onset of the pandemic is well recognized for its overall decline in emergency department consultations and decreased trauma and fracture incidence, the impact of the pandemic on fracture incidence by fracture type and age of individuals has largely remained unclear until now. The present study found a substantial decline in nearly all fracture types among younger individuals, with significant decreases, particularly in fracture types typically related to sports injuries or high-impact trauma. The most significant decline was observed in fibula fractures following the onset of COVID-19, with a total decrease of 66.68%. This finding aligns with the literature, as fibula fractures and ankle sprains primarily occur in middle-aged patients, with falls and sports injuries accounting for up to 75% of the cases [5,7].

Government-imposed restrictions to contain the spread of the COVID-19 virus have led to a considerable downturn in team and community sports. Schneider and colleagues noted a significant reduction in sporting and physical activities among athletes who typically train in a club environment [8]. These athletes also reported dedicating less time to sports activities in the same study [8].

Moreover, a well-documented shift has occurred during the COVID-19 pandemic, with individuals reducing their participation in sports and physical leisure activities in exchange for home-based and digital/online activities [9,10,11]. This change in the activity patterns of young and mid-aged individuals correlates strongly with the noted decrease in sports-related fractures, particularly in the case of distal fibula fractures [12]. Additionally, pelvic, rib, and femoral shaft fractures are commonly associated with high and severe impact traumas, such as road traffic accidents (RTA) [13,14], particularly in the young and mid-aged population [15]. In the post-outbreak period, there was a 10.96% decrease in RTAs compared to the average pre-outbreak period in Germany. This situation could account for the observed 15.31% reduction in pelvic fractures and a 12.62% decrease in rib fractures following the onset of COVID-19. The decline in RTAs and related fractures can likely be attributed to the implemented isolation measures, which led to a substantial decrease in mobility and global transportation [16].

While there was a consistent and uniform decline in fracture incidence among younger individuals, fracture incidence among older individuals showed a twofold change depending on the type of fracture. The fracture incidence of hip fractures, commonly referred to as fragility fractures (femoral neck, subtrochanteric, and pertrochanteric femoral fractures), remained mainly unchanged, while at the same time, non-fragility fractures showed a significant decline well below the expected 99% prediction intervals. To date, only a handful of studies have explored the incidence of fragility fractures during the COVID-19 pandemic, and their results have been varied and inconsistent. Some research suggests a considerable decrease in the overall fracture incidence following the onset of COVID-19, yet the rate of fragility fractures in the hip and spine among elderly patients remains steady [17,18,19]. Conversely, other studies have suggested a significant decrease in fragility fractures [20,21,22]. The unchanged or even escalating number of fragility fractures, particularly around the hip, during the COVID-19 pandemic, appears to stem from multiple factors. Firstly, fragility fractures of the proximal femur seldom correlate with high-impact trauma or intense physical activity; thus, they are less likely to be influenced by the stay-at-home orders issued during the pandemic. In fact, over half of these fractures occur indoors, predominantly due to low-energy traumas [23]. Secondly, the scope of the pandemic further extends to bone metabolism. During the pandemic, a substantial drop in the treatment of apparent osteoporosis and adherence to medication has been seen, thereby increasing the likelihood of non-traumatic fractures due to further bone demineralization [24]. The overall reduction in medical consultations during the pandemic has led to significant delays and decreases in the diagnosis and treatment of osteoporosis [25]. Additionally, the COVID-19 infection has been linked to a loss of trabecular mass through overexpression of osteoclasts, as seen in mouse models [26].

At the same time, the COVID-19 pandemic may have significantly altered factors that influence the risk of falling in the elderly. Research has shown that infection with the new coronavirus may display such symptoms as dizziness and confusion in the elderly, thus increasing the risks of falls [27]. Moreover, the pandemic has also caused a deep disruption in common social activities and gatherings with the isolation and stay-at-home measures. This, in turn, has evoked a sharp increase in loneliness and a feeling of anxiety in the elderly population, which has been identified as a major risk factor for falls [28,29]. Moreover, the lockdown has led to significant changes in people’s activity profiles, notably an increase in home-based and digital/online leisure activities, resulting in a further decrease in physical activity in the elderly population [11]. The impact and repercussions of reduced physical activity are well-known with regard to the increased risk of falls and decline in bone metabolism [30,31,32]. To the best of our knowledge, this is the very first study reporting on fracture incidence during the COVID-19 pandemic on a nationwide basis. So far, studies investigating the fracture rate with the onset of the pandemic have been based mainly on a single-center analysis of local or regional hospitals, increasing the risk of under- or overestimating the real association of the COVID-19 pandemic with the fracture incidence.

## 5. Conclusions

The onset of the COVID-19 pandemic had a significant impact on the observed fracture incidence. Individuals < 65 years showed a uniform decrease in fracture incidence when compared with the pre-COVID-19 period. Among older individuals (> 65 years), the incidence of fragility fractures remained mainly unchanged, while non-fragility fractures displayed a sharp and significant decrease in incidence with the onset of the pandemic.

## Figures and Tables

**Table 1:** Change in the average number of fractures before and after the onset of COVID-19 (2010–2019 and 2020–2021, respectively). The data are supplementary to Figure 1.

Fracture Type	Change in the Mean Fracture Number Per Year for the COVID-19 Pre-Outbreak vs. Post-Outbreak Period		*p*-Value	
**Age**	**18–64**	**>65**	**18–64**	**>65**
Femoral neck	+4.02%	+10.59%	0.24	0.03
Pertrochanteric	−2.21%	+9.68%	0.24	0.05
Distal radius	−2.65%	−6.61	0.62	0.24
Proximal humerus	−4.48%	+0.25%	0.03	0.91
Lumbal spine and pelvis	−15.32% *	−1.59%	<0.01	0.84
Fibula	−66.68%	−68.61%	0.29	0.28
Cervical spine	−3.29%	+21.91%	0.43	0.12
Humeral shaft	−11.53% *	+4.71%	<0.01	0.32
Subtrochanteric	+1.07%	+14.32%	0.69	0.04
Femoral shaft	−19.21% *	+22.13%	<0.01	0.04
Clavicle	+2.24%	+16.59%	0.73	0.18
Thoracic spine	−17.78% *	+2.02%	<0.01	0.79
Ribs	−12.62% *	−1.00%	<0.01	0.89
Proximal tibia	−6.70% *	+0.80%	<0.01	0.83

*: 18–64 vs. >65; *t*-test; *p* ≤ 0.01.

**Table 2:** Mean annual number of fractures from pre-outbreak to post-outbreak period.

Cases Per Year (×1000)	Female 18–64 yr.		Female > 65 yr.		Male 18–64 yr.		Male > 65 yr.	
	**Pre-Outbreak**	**Post-Outbreak**	**Pre-Outbreak**	**Post-Outbreak**	**Pre-Outbreak**	**Post-Outbreak**	**Pre-Outbreak**	**Post-Outbreak**
**Femoral neck**								
mean (med)	4.49 (4.47)	4.69 (4.69)	46.36 (46.39)	50.00 (50.00)	4.39 (4.37)	4.56 (4.56)	18.95 (19.01)	23.04 (23.04)
[min–max]	[4.26–4.86]	[4.66–4.71]	[43.81–49.81]	[49.67–50.33]	[4.1–4.64]	[4.54–4.59]	[16.2–22.36]	[22.43–23.65]
**Pertrochanteric**								
mean (med)	2.24 (2.24)	2.25 (2.25)	45.77 (46.13)	49.1 (49.1)	3.64 (3.63)	3.5 (3.5)	15.97 (16.11)	19.25 (19.25)
[min–max]	[2.14–2.33]	[2.22–2.27]	[42.88–49.16]	[48.91–49.29]	[3.47–3.79]	[3.47–3.53]	[13.68–18.69]	[18.9–19.61]
**Distal radius**								
mean (med)	23.86 (23.77)	24.03 (24.03)	42.69 (41.76)	39.93 (39.93)	13.06 (12.9)	11.94 (11.94)	4.98 (4.9)	4.79 (4.79)
[min–max]	[21.45–29.26]	[22.66–25.4]	[40.26–49.52]	[38.02–41.83]	[12.68–13.88]	[11.78–12.09]	[4.57–5.94]	[4.54–5.04]
**Proximal humerus**								
mean (med)	10.08 (10.14)	10.03 (10.03)	34.67 (34.69)	34.31 (34.31)	6.88 (6.85)	6.2 (6.2)	7.73 (7.77)	8.19 (8.19)
[min–max]	[9.59–10.45]	[9.89–10.18]	[33.74–36.14]	[33.8–34.83]	[6.61–7.32]	[6.01–6.39]	[7.08–8.49]	[7.94–8.44]
**Lumbar spine and pelvis**								
mean (med)	8.03 (8.06)	7.01 (7.01)	43.39 (44.36)	41.55 (41.55)	10.15 (10.18)	8.76 (8.76)	16.34 (16.63)	17.25 (17.25)
[min–max]	[7.64–8.28]	[6.93–7.09]	[36.68–47.49]	[41.11–41.99]	[9.75–10.35]	[8.59–8.92]	[13.42–18.73]	[17.21–17.29]
**Fibula**								
mean (med)	17.91 (12.6)	11.68 (11.68)	12.24 (8.34)	6.96 (6.96)	18.35 (13.23)	10.08 (10.08)	5.16 (4.03)	3.36 (3.36)
[min–max]	[11.9–36.12]	[10.61–12.74]	[7.67–23.47]	[6.75–7.17]	[12.09–34.99]	[9.3–10.85]	[3.54–9.24]	[3.22–3.5]
**Rib**								
mean (med)	3.01 (3.05)	2.68 (2.68)	12.6 (13.01)	12.11 (12.11)	9.56 (9.51)	8.48 (8.48)	10.98 (11.27)	11.24 (11.24)
[min–max]	[2.76–3.15]	[2.67–2.69]	[10.8–13.73]	[11.97–12.24]	[9.26–9.81]	[8.31–8.65]	[9.06–12.58]	[11.23–11.25]
**Thoracic spine**								
mean (med)	3.62 (3.64)	3.01 (3.01)	13.25 (13.36)	12.93 (12.93)	4.81 (4.81)	4.14 (4.14)	4.79 (4.80)	5.48 (5.48)
[min–max]	[3.40–3.83]	[3.01–3.02]	[11.59–14.54]	[12.72–13.14]	[4.71–4.89]	[4.10–4.18]	[3.72–5.70]	[5.38–5.59]
**Clavicula**								
mean (med)	3.45 (3.45)	3.69 (3.69)	2.4 (2.35)	2.78 (2.78)	13.28 (13.63)	13.43 (13.43)	1.67 (1.67)	2.11 (2.11)
[min–max]	[2.77–3.93]	[3.53–3.85]	[1.7–3.08]	[2.76–2.79]	[10.79–14.56]	[12.76–14.09]	[1.18–2.21]	[2.09–2.14]
**Proximal tibia**								
mean (med)	5.82 (5.77)	6.10 (6.10)	5.26 (5.19)	5.35 (5.35)	5.89 (5.85)	4.88 (4.88)	1.56 (1.53)	1.52 (1.52)
[min–max]	[5.23–6.49]	[5.97–6.23]	[5–5.72]	[5.33–5.38]	[5.66–6.11]	[4.7–5.05]	[1.43–1.75]	[1.5–1.54]
**Femoral shaft**								
mean (med)	1.15 (1.14)	1.04 (1.04)	6.71 (6.42)	8.40 (8.40)	2.36 (2.35)	1.90 (1.90)	2.00 (1.89)	2.80 (2.80)
[min–max]	[1.08–1.21]	[1.04–1.04]	[5.48–8.21]	[8.21–8.58]	[2.17–2.59]	[1.84–1.97]	[1.52–2.69]	[2.74–2.85]
**Subtrochanteric**								
mean (med)	0.59 (0.59)	0.65 (0.65)	7.89 (7.7)	8.89 (8.89)	1.00 (0.98)	0.95 (0.95)	2.69 (2.63)	3.46 (3.46)
[min–max]	[0.54–0.65]	[0.62–0.68]	[7.2–8.86]	[8.88–8.9]	[0.95–1.06]	[0.91–1.00]	[2.2–3.34]	[3.4–3.52]
**Humeral shaft**								
mean (med)	2.09 (2.10)	1.98 (1.98)	5.78 (5.8)	6.04 (6.04)	2.29 (2.26)	1.94 (1.94)	1.67 (1.64)	1.79 (1.79)
[min–max]	[2.01–2.15]	[1.93–2.03]	[5.38–6.39]	[5.96–6.11]	[2.11–2.55]	[1.86–2.03]	[1.46–1.9]	[1.78–1.79]
**Cervical spine**								
mean (med)	0.78 (0.80)	0.73 (0.73)	3.72 (3.85)	4.50 (4.50)	2.02 (2.02)	1.98 (1.98)	2.92 (2.98)	4.00 (4.00)
[min–max]	[0.69–0.84]	[0.7–0.77]	[2.4–4.73]	[4.4–4.6]	[1.8–2.14]	[1.89–2.07]	[1.86–3.99]	[3.96–4.05]

Heatmap illustrating the mean annual incidence (×1000 cases) of fractures between the pre-outbreak and post-outbreak periods (2010–2019 and 2020–2021, respectively). The mean (mean), median (med), minimum (min), and maximum (max) values are given. Statistical analysis was performed using the *t*-test. The color scheme denotes green cells for statistically significant increase (0.01 < *p* < 0.05), brown cells for significant decrease (*p* ≤ 0.01), and yellow cells for a significant decrease (0.01 < *p* < 0.05) between the pre-outbreak and post-outbreak periods.

**Table 3:** Number of fractures between 2018 and 2019 and 2020 and 2021.

Cases Per Year (×1000)	All Genders >18	Female 18–64 yr.	Female >65 yr.	Male 18–64 yr.	Male >65 yr.
**Femoral neck**					
2018 and 2019	79.33 and 81.59	4.68 and 4.86	48.35 and 49.81	4.61 and 4.56	21.70 and 22.36
2020 and 2021	81.35 and 83.24	4.71 and 4.66	49.67 and 50.33	4.54 and 4.59	22.43 and 23.65
**Pertrochanteric**					
2018 and 2019	71.89 and 73.8	2.32 and 2.33	47.88 and 49.16	3.62 and 3.62	18.06 and 18.69
2020 and 2021	73.51 and 74.69	2.22 and 2.27	48.91 and 49.29	3.47 and 3.53	18.90 and 19.61
**Distal radius**					
2018 and 2019	84.10 and 82.16	23.86 and 23.37	41.84 and 41.05	13.44 and 12.83	4.95 and 4.91
2020 and 2021	77.31 and 84.06	22.66 and 25.40	38.02 and 41.83	12.09 and 11.78	4.54 and 5.04
**Proximal humerus**					
2018 and 2019	60.28 and 61.67	10.22 and 10.24	35.33 and 36.14	6.61 and 6.80	8.11 and 8.49
2020 and 2021	58.01 and 59.45	9.89 and 10.18	33.80 and 34.83	6.39 and 6.01	7.94 and 8.44
**Lumbar spine and pelvis**					
2018 and 2019	84.61 and 83.71	8.24 and 8.22	47.49 and 47.09	10.15 and 9.75	18.73 and 18.64
2020 and 2021	74.41 and 74.73	7.09 and 6.93	41.11 and 41.99	8.92 and 8.59	17.29 and 17.21
**Fibula**					
2018 and 2019	36.38 and 35.70	12.18 and 12.04	8.11 and 7.67	12.33 and 12.09	3.76 and 3.92
2020 and 2021	29.88 and 34.26	10.61 and 12.74	6.75 and 7.17	9.30 and 10.85	3.22 and 3.50
**Rib**					
2018 and 2019	38.75 and 38.92	3.08 and 3.1	13.54 and 13.73	9.69 and 9.5	12.44 and 12.58
2020 and 2021	34.79 and 34.21	2.67 and 2.69	12.24 and 11.97	8.65 and 8.31	11.23 and 11.25
**Thoracic spine**					
2018 and 2019	28.43 and 28.50	3.66 and 3.53	14.31 and 14.54	4.85 and 4.73	5.62 and 5.70
2020 and 2021	25.28 and 25.85	3.01 and 3.02	12.72 and 13.14	4.18 and 4.10	5.38 and 5.59
**Clavicula**					
2018 and 2019	23.38 and 23.62	3.84 and 3.93	2.89 and 3.08	14.56 and 14.39	2.09 and 2.21
2020 and 2021	22.79 and 21.21	3.85 and 3.53	2.76 and 2.79	14.09 and 12.76	2.09 and 2.14
**Proximal tibia**					
2018 and 2019	19.22 and 19.62	6.28 and 6.49	5.60 and 5.72	5.70 and 5.66	1.63 and 1.75
2020 and 2021	18.19 and 17.50	6.23 and 5.97	5.38 and 5.33	5.05 and 4.70	1.54 and 1.50
**Femoral shaft**					
2018 and 2019	13.91 and 14.15	1.13 and 1.08	7.94 and 8.21	2.29 and 2.17	2.55 and 2.69
2020 and 2021	13.96 and 14.31	1.04 and 1.04	8.21 and 8.58	1.97 and 1.84	2.74 and 2.85
**Subtrochanteric**					
2018 and 2019	13.52 and 13.83	0.61 and 0.65	8.76 and 8.86	0.95 and 0.98	3.21 and 3.34
2020 and 2021	13.95 and 13.95	0.68 and 0.62	8.88 and 8.90	1.00 and 0.91	3.40 and 3.52
**Humeral shaft**					
2018 and 2019	12.35 and 12.50	2.12 and 2.10	6.19 and 6.39	2.24 and 2.11	1.80 and 1.90
2020 and 2021	11.67 and 11.81	1.93 and 2.03	6.11 and 5.96	1.86 and 2.03	1.78 and 1.79
**Cervical spine**					
2018 and 2019	11.35 and 11.56	0.84 and 0.80	4.55 and 4.73	2.14 and 2.04	3.82 and 3.99
2020 and 2021	11.19 and 11.24	0.77 and 0.70	4.40 and 4.60	2.07 and 1.89	3.96 and 4.05

Heatmap illustrating the annual incidence (×1000 cases) of fractures between 2018 and 2019 and 2020 and 2021. Statistical analysis was performed using the *t*-test. The color scheme denotes brown cells for a significant difference (*p* ≤ 0.01) and yellow cells for a significant difference between 0.01 < *p* < 0.05 between the periods 2018 and 2019 and 2020 and 2021.

## Data Availability

Upon a reasonable request, we are open to sharing our data with anyone who is interested.
